# Early childhood risk and resilience factors for behavioural and emotional problems in middle childhood

**DOI:** 10.1186/1471-2431-14-166

**Published:** 2014-07-01

**Authors:** Jason L Cabaj, Sheila W McDonald, Suzanne C Tough

**Affiliations:** 1 Department of Community Health Sciences, University of Calgary, Calgary, Alberta, Canada; 2 Department of Pediatrics, University of Calgary, Calgary, Alberta, Canada

## Abstract

**Background:**

Mental disorders in childhood have a considerable health and societal impact but the associated negative consequences may be ameliorated through early identification of risk and protective factors that can guide health promoting and preventive interventions. The objective of this study was to inform health policy and practice through identification of demographic, familial and environmental factors associated with emotional or behavioural problems in middle childhood, and the predictors of resilience in the presence of identified risk factors.

**Methods:**

A cohort of 706 mothers followed from early pregnancy was surveyed at six to eight years post-partum by a mail-out questionnaire, which included questions on demographics, children’s health, development, activities, media and technology, family, friends, community, school life, and mother’s health.

**Results:**

Although most children do well in middle childhood, of 450 respondents (64% response rate), 29.5% and 25.6% of children were found to have internalising and externalising behaviour problem scores in the lowest quintile on the NSCLY Child Behaviour Scales. Independent predictors for problem behaviours identified through multivariable logistic regression modelling included being male, demographic risk, maternal mental health risk, poor parenting interactions, and low parenting morale. Among children at high risk for behaviour problems, protective factors included high maternal and child self-esteem, good maternal emotional health, adequate social support, good academic performance, and adequate quality parenting time.

**Conclusions:**

These findings demonstrate that several individual and social resilience factors can counter the influence of early adversities on the likelihood of developing problem behaviours in middle childhood, thus informing enhanced public health interventions for this understudied life course phase.

## Background

The public health burden of childhood mental and behavioural problems is substantial. The point prevalence of mental disorders in youth has been estimated to be between 10% and 20%, with even higher rates found in disadvantaged children [1-3]. Further, because childhood behaviour exists on a continuum, many children that do not meet criteria for clinical diagnoses still exhibit maladaptive emotional and behavioural traits that have a substantial influence on long-term outcomes in multiple domains, including academic achievement, health, and social and economic success [4]. Notably, the origins of mental illnesses that persist throughout the lifecycle often have their origins in childhood, manifesting as both internalizing and externalizing behaviours. In Canada, although mental health spending is lower than in most developed countries, more than $14 billion in government expenditures went towards mental health in 2010 [5]. When health related quality-of-life losses are considered, the economic burden of mental disorders in Canada has been estimated to exceed $50 billion per year [6].

A growing body of research suggests that developmental trajectories resulting in poor health outcomes are established early in life and are predicted by numerous prenatal, perinatal, and childhood factors that reflect environmental adversity [7-9]. Exposure to misfortune in early childhood has been shown to increase the odds of poor mental health and problem behaviours that persist into adolescence and adulthood, such as antisocial tendencies, substance abuse, mood disorders, and suicide attempts [10-13]. In some cases, a dose response relationship between the number of adverse childhood events experienced and later mental health problems has been demonstrated [14].

Because few young people are entirely free from risk, and the existing options for treatment of children and adolescents diagnosed with mental disorders remain limited, the further development of effective preventive approaches would have enormous potential benefits [15,16]. Thus, as Waddel et al. and others have noted, determination of both the risk factors associated with mental disorders, *and* the protective factors which may either lower the likelihood or reduce the negative impact of these outcomes, is necessary to inform the planning and implementation of preventive and health promoting interventions [17,18]. Modifiable protective factors that have been identified in previous research include parenting practices and levels of confidence, social support, and maternal mental health [19-22].

This paper describes the most recent study arising from a cohort of mothers and children in Calgary, an urban centre in Alberta, Canada, that have been followed since the perinatal period and surveyed periodically (at three, five, and now eight years of age). The first Community Perinatal Care Study, a randomized controlled trial (RCT) of three types of prenatal care, found that additional prenatal support from nurses and home visitors increased the use of community based resources and access to pregnancy-related information, but did not alter alcohol/tobacco use, post-partum depression, or birth outcomes [23]. A follow-up survey at three years of age reported that 11% of this demographically low-risk (by maternal education and family income) sample of Canadian children screened at high risk for developmental problems, with poor maternal mental health identified as the strongest predictor of a positive screen. [24]. Subsequently, follow-up of the cohort at age five identified maternal well-being and history of abuse as primary risk factors for developmental problems, and documented the persistent influence of maternal influences on infant and child development up to school entry [25].

The third Community Perinatal Care (CPC-8) follow-up study, called “It’s All about Me! Middle Childhood Survey”, was designed to explore family, school and community life of children through a questionnaire distributed in middle childhood. The objectives of the present study were to use CPC-8 data to identify the combination of current and past demographic, familial and environmental factors associated with emotional or behavioural problems in middle childhood, and the predictors of resilience in the presence of previously identified risk factors for delayed development. We hypothesized that adversity in early and middle childhood would be associated an increased risk of internalizing and externalizing behaviours, but that enhanced social and emotional well-being could provide protection against poor mental health outcomes.

## Methods

### Participants

The participants in this study are part of the longitudinal Community Perinatal Care (CPC) cohort that had been followed since pregnancy [23]. The initial sample for the CPC study included pregnant women over 18 years of age who attended one of three family physician low-risk maternity practices in the Calgary Health Region. Mothers who agreed to participate beyond the randomized controlled trial were surveyed as part of the first follow up study (CPC-3) when their children were three years old (n = 791). Subsequently, when the children were aged four to six years and six to eight years respectively, participants from the CPC-3 study that indicated willingness to participate in future research formed the cohorts for both the second (CPC-5) and third (CPC-8) follow-up studies. Exclusion criteria consisted of the inability to complete the questionnaire in English and lack of current mailing information after exhaustive searching. Findings from the original CPC study and the first two follow-up studies are reported elsewhere [23-25].

### Questionnaire

The CPC-8 survey (consisting of a 21-page questionnaire) included questions on demographics, children’s health, development, activities, media and technology, family, friends, community, school life, and mother’s health (see Additional file 1). The questionnaire was revised based on pilot testing with a small sample of mothers (n = 13) for length, flow, comprehension, and response burden, and took about 20–25 minutes to complete.

Postcards outlining plans for another CPC follow-up study were mailed to the last known address of the 706 respondents from the CPC-3 study in the summer of 2009. Research assistants then used Facebook, directory assistance, and study database phone numbers to contact respondents whose postcards had been returned-to-sender. In January 2010, the CPC-8 questionnaire was sent to these mothers along with a cover letter informing participants of the voluntary nature of their participation, confidentiality of their information, and a description of potential linkages with previously collected data. Mothers also received a postage-paid envelope, and a one-time recreation pass (in appreciation of their time and contribution to the study). The methods described above were again used by research assistants to obtain updated addresses when study questionnaires were returned-to-sender.

Reminder phone calls were made at one and two months after the survey mail-out to mothers with outstanding questionnaires, and letters were sent at 3 months to women who could not be contacted by phone reminding them of the study and requesting they call study investigators if they required another copy of the questionnaire. A second copy of the questionnaire was sent to women who had expressed a commitment to return the questionnaire and to those who research assistants had not been able to speak with on the phone. Finally, further phone calls were made to mothers with outstanding questionnaires that had received a second copy and/or had expressed intent to complete the questionnaire. Data collection and follow-up ended in June 2010. Questionnaires were scanned to an Access database after verification with Teleform, an electronic data capture and management system [26]. Ethics approval was granted to the study from the Conjoint Health Research Ethics Board at the University of Calgary.

### Variables

Study variables, including dependent and independent variables, were drawn from all data collection time points for the CPC cohort. Both single item investigator derived questions and standardized instruments were used.

### Outcome measures

Study outcome variables were problem behaviours, a classification intended to capture a range of perceived difficulties in children and adolescents (i.e. medical, biological, and psychological conditions). The specific outcomes assessed were the presence of externalizing behaviours, in which psychosocial maladjustment is manifested outwardly (e.g. hyperactivity, aggression, or violence), and internalizing behaviours, in which distress is manifested in an inhibited style of social interaction (e.g. such as anxiety or depression). Outcomes were measured using the National Longitudinal Survey of Children and Youth (NLSCY) Child Behavioural Scales [27], which were derived from a pool of items from previous studies and underwent psychometric testing to ensure validity with DSM-IV criteria [28]. Scales that assessed externalizing and internalizing behaviours were combined to produce externalizing and internalizing dimensions, respectively. For subscales that composed the externalizing dimension, Chronbach’s alpha reliability coefficient ranged from 0.77-0.84. The reliability coefficient for the internalizing scale was 0.79. For each dimension, scores at or above the 80th percentile of the distribution were used to classify children as manifesting problem behaviours, consistent with prior studies using these scales [29].

### Independent variables

Predictor variables fell into three groups: demographic factors, child characteristics, and maternal characteristics.

#### Demographic factors

Demographic information based on maternal self-report collected in CPC studies included marital status, education, annual household income, ethnicity, and household composition. Indicator variables to capture demographic risk were derived for both age three (at least one of: single marital status, less than 25 years old, less than a high school education, household income less than $40,000, or moved two or more times in the past two years), and age eight (at least one of: single marital status; household income less than $40,000; not enough money for food and daily living expenses in the past 3 months; visited food bank in the past 3 months; or not able to pay all of their bills in the past 3 months).

Although we strove for consistency in defining demographic risk, the definition of historical and current demographic risk changed slightly across time due to the availability and relevancy of the variables collected at each time point. For example, young maternal age was included in history of demographic risk but was no longer relevant when the child approached age 8. Despite this, our demographic risk variables captured constructs of socioeconomic status and indicators of vulnerability (residential stability and food insecurity) at each time point.

#### Child characteristics

Child gender, health status, body mass index, history of specialist referral, school performance, and history of stressful or traumatic childhood events were collected based on maternal report in CPC-8. Information from the Parents’ Evaluation of Developmental Status (PEDS) standardized measurement scale collected in CPC-3 and CPC-5 follow-up studies was used to determine risk of developmental disability [30].

#### Maternal characteristics

Information on maternal physical and emotional health status, (excellent, good, fair, poor, or terrible) [31], history of abuse (any abuse prior to pregnancy), and adequacy of social support were based on self-report data collected during pregnancy, at age three, and at age eight. Information about parenting collected included parenting morale, assessed using the Parenting Morale Index [32] at 3 years post-birth, and parenting style, which was assessed using two subscales of the National Longitudinal Survey of Children and Youth (hostile/ineffective and aversion) [33] at the middle childhood follow-up.

Maternal mental health risk indicators were developed to describe risk during pregnancy (at least one of: abuse prior to pregnancy or up to 6–8 weeks postpartum, depression prior to pregnancy, suicidal thoughts prior to pregnancy, poor social support in first trimester, poor network orientation in first trimester, or poor emotional health in first trimester), at age three (at least one of: experience of abuse since child was born, depression for six or more months after giving birth, poor social support, or poor emotional health), and age eight (at least one of: low social support, poor emotional health, or unstable spouse/partner events in the past 12 months).

### Analysis

Data collected in the CPC-8 questionnaire was linked to that from the original CPC study as well as to the previous follow up studies through unique study identification numbers. Data were analysed using the statistical package SPSS (v.19). Data analysis included descriptive methods for categorical and continuous variables as well as bivariate and multivariable methods. For continuous predictors and predictor variables with greater than two levels, dichotomization was carried out for ease of interpretation based on the theoretically most meaningful categories and consistent with previous work using CPC data [24,25]. For each outcome, we identified at least 5 variables from each previous and current time point that were significant at p < 0.01 in bivariate analysis. This provided a range of both previous and current risk factors covering child and maternal domains for inclusion in the multivariable analysis.

We developed a predictive model for each behavioural dimension using a manual stepwise model building approach that considered current (age 8) risk factors in the first block, followed by incorporation of previous risk factors (age 3 and age 5), to produce a final, parsimonious model. This approach allowed for the assessment of the independent effects of current influences while accounting for risk factors that occurred earlier in childhood. Predictor variables were included in the regression models if they were significantly associated with the outcome in bivariate analysis (using Chi Square or Fisher’s exact test) at p ≤ 0.01, or there was theoretical rationale (i.e. gender and demographic risk were included in the models regardless of significance of the association with the outcome variables).

To assess resilience in the presence of previously identified risk, a subsample of mothers was selected from the broad study population based on having either demographic or mental health risk (as defined above) when their child was three years old. In order to compare those at the highest risk to those at the lowest risk of problem behaviours, the internalizing and externalizing behaviour scores were categorized at the ‘extremes’ to capture children who scored either high (80th percentile and above) or low (20th percentile and below) for each dimension. Chi square analysis was carried out to assess the influence of potential protective factors that discriminated children scoring in the low vs. high externalizing or internalizing behaviour categories.

## Results

### Demographics

**Figure 1:**
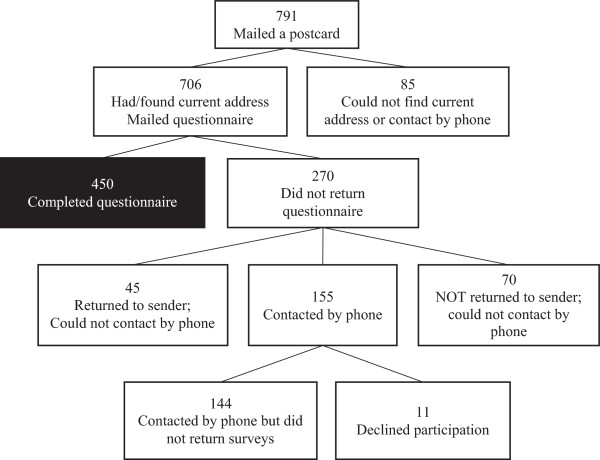
Flowchart of eligibility, recruitment, and completion of mothers who participated in the CPC-8 follow-up study.

Of the 706 eligible participants, 450 returned the questionnaires, leading to a 64% response rate (Figure 1). The majority of mothers who participated in the study were white/Caucasian (87.6%), married (93.6%), had completed a post-secondary education (74.2%), and had a household income of at least $60,000 (88.4%) (Table 1). The average age of these women was 38.4 years (SD = 4.48) and 66.2% reported that one or two children lived in the household. Twelve percent of mothers reported having a history of demographic risk at age three, while at age eight, approximately one-quarter of the sample reported demographic risk.

**Table 1:** Characteristics of mothers and children who participated in the CPC-8 study

**Characteristic**	**Total sample (N = 450) n (%)**
**Demographics**	
Maternal age*	
Mean (SD)	38.42 (4.478)
Marital status*	
Married/common-law	421 (93.6)
Single, divorced, or separated	29 (6.4)
Education*	
Completed post-secondary	334 (74.2)
Less than post-secondary	116 (25.8)
Household income*	
Less than $60,000	52 (11.6)
$60,000 or more	397 (88.4)
Ethnicity**	
White/Caucasian	394 (87.6)
Other	56 (12.4)
Number of children in household*	
One to two children	298 (66.2)
Three or more children	152 (33.8)
History of demographic risk: less than 25 years old, less than $40,000 income, single, high school education or less, or moved 2 or more times in the past 2 years**	
Yes	55 (12.2)
No	395 (87.8)
Current demographic risk: less than $40,000 income, single, or food/expense instability*	
Yes	110 (24.5)
No	339 (75.5)
**Child Characteristics**	
Gender**	
Girl	228 (52.2)
Boy	209 (47.8)
Preterm infant**	
Yes	22 (5.0)
No	414 (95.0)
General health*	
Excellent, very good, or good	441 (98.2)
Fair or poor	8 (1.8)
BMI status*	
Underweight	39 (9.7)
Healthy	297 (74.1)
Overweight/obese	65 (16.2)
Number of health problems as told by health professional*	
No health problems	278 (61.8)
At least one health problem	172 (38.2)
High externalizing behaviour (scored ≥ 80^th^ percentile)*	
Yes	114 (25.6)
No	332 (74.4)
High internalizing behaviour (scored ≥ 80^th^ percentile)*	
Yes	132 (29.5)
No	315 (70.5)
Low prosocial behaviour (scored ≤ 20^th^ percentile)*	
Yes	116 (25.9)
No	332 (74.1)
Referral to at least one of early intervention program, speech or language pathologist, developmental pediatrician, psychologist, physiotherapist, or dietician at 3 years or 5 years**	
Yes	123 (27.3)
No	327 (72.7)
PEDS Path A at 3 years or 5 years of age**	
Yes	83 (21.8)
No	297 (78.2)
**Maternal Characteristics**	
History of mental health risk: abuse (prior to pregnancy, during pregnancy, 6–8 weeks postpartum), depression (prior to pregnancy), suicide (prior to pregnancy), poor social support (first trimester), poor network orientation (first trimester), poor emotional health (first trimester)**	
Yes	211 (46.9)
No	239 (53.1)
Mental health risk at CPC3Year: abuse, depression (6+ months postpartum), poor social support, poor emotional health**	
Yes	81 (18.0)
No	369 (82.0)
Current mental health risk: low social support, poor emotional health, or unstable spouse/partner events in the past 12 months*	
Yes	138 (31.2)
No	305 (68.8)
Low positive parenting interaction (scored ≤ 20^th^ percentile)*	
Yes	128 (28.4)
No	322 (71.6)
Low social support (scored <15^th^ percentile)*	
Yes	48 (10.7)
No	399 (89.3)
Emotional health in past 6 months*	
Excellent, very good, or good	382 (84.9)
Fair or poor	68 (15.1)
Unstable events that occurred during the past 12 months to mother or spouse/partner*	
None to two events	370 (83.5)
Three or more events	73 (16.5)

*variable assessed at current (middle childhood) assessment time point.

**variable assessed at previous time points.

### Child and maternal characteristics

The children in the study were evenly distributed based on gender (48.7% male), with 5.0% being preterm infants. The majority of mothers reported their children having above average general health (98.2%), a healthy BMI (74.1%), and no health problems as told by a health professional (61.8%) at age 8. According to maternal self-report for child behaviour, 29.5% and 25.6% of children were found to have internalising and externalising behaviour problem scores, respectively, in the lowest quintile of the distribution on the NSCLY Child Behaviour Scales. At either age 3 or 5, 21.8% of children had screened at high risk of developmental disabilities (Path A) according to the Parents’ Evaluation of Development, and 27.3% of children had a history of referral for any developmental or behavioural concern.

Nearly half of the mothers reported a history of mental health risk during pregnancy (46.9%). When their children were three years of age, mental health risk was observed in 18.0% of the sample, while mental health risk at age eight was seen in 31.2% of mothers. The different proportions seen according to timing of assessment can be attributed to the different elements that are included in each definition of ‘risk’, and are largely a reflection of questions asked at the different time points. This is important to keep in mind when interpreting these results, as mental health risk is differentially defined across time. The majority of mothers reported positive parenting interaction with their children (71.6%), adequate social support (89.3%)**,** and above average emotional health (84.9%) when their children were 8.

### Key factors associated with internalizing and externalizing behaviours

Observed risk factors for internalizing behaviour problems at age eight included being male (OR: 1.70; 95% CI: 1.02, 2.82), previous demographic risk at age 3 (OR: 2.82; 95% CI: 1.27, 6.26), current maternal mental health risk (OR: 1.96; 95% CI: 1.15, 3.36), current low positive parenting interaction (OR: 1.92; 95% CI: 1.12, 3.30), and previous low parenting morale (OR: 2.62; 95% CI: 1.43, 4.82) (Table 2).

**Table 2:** Key risk factors for problem behaviours at age eight

**Variable**	**Externalizing behaviours**		**Internalizing behaviours**	
	**Adjusted odds ratio (95% C.I.)**	**p-value**	**Adjusted odds ratio (95% C.I.)**	**p-value**
Male gender	2.64 (1.50, 4.65)	0.001	1.70 (1.02, 2.82)	0.042
Current demographic risk	1.63 (0.86, 3.10)	0.134		
Past demographic risk			2.82 (1.27, 6.26)	0.011
Current maternal mental health risk			1.96 (1.15, 3.36)	0.014
Past maternal mental health risk	2.02 (1.02, 4.01)	0.044		
Absence of positive parenting interactions			1.92 (1.12, 3.30)	0.017
Past low parental sense of competence	2.83 (1.58, 5.06)	<0.001		
Past hostile parenting style	2.24 (1.12, 4.50)	0.023		
Past low parenting morale			2.62 (1.43, 4.82)	0.002
Past unhappy events for child	2.14 (1.09, 4.19)	0.027		
Developmental referral history	1.99 (1.04, 3.83)	0.039		
Poor school performance	2.07 (1.16, 3.69)	0.014		

*Note*. Internalizing and externalizing behaviours defined by behaviour scales previously used in the Ontario Child Health Study (OCHS) with the cut-offs made at the 80th percentile (coding: Less than 80th percentile vs. 80th percentile or better).

For externalizing behaviours, being male (OR: 2.64; 95% CI: 1.50, 4.65), previous maternal mental health risk at age 3 (OR: 2.02; 95% CI: 1.02, 4.01), previous hostile parenting at age 3 (OR: 2.24; 95% CI: 1.12, 4.50), previous low satisfaction in parenting sense of competence at age 5 (OR: 2.83; 95% CI: 1.58, 5.06), previous referral for developmental or behavioural concerns at age 3 (OR: 1.99; 95% CI: 1.04, 3.83), any unhappy childhood event (OR: 2.14; 95% CI: 1.09, 4.19), and current poor to average school performance (OR: 2.07; 95% CI: 1.16, 3.69) were independent risk factors (Table 2).

### Factors related to positive outcomes in the presence of previous risk

Among mothers with previously identified demographic or mental health risk when their child was 3 years old (n = 111) [24], a low degree of internalizing behaviours in the child was associated with high overall self-esteem at age 8 as reported by the mother (89.5 vs. 63.3%; p = 0.033). Child factors associated with a low degree of externalizing behaviours included mothers report that their 8-year old child had two or more close friends (93.8 vs. 72.1%; p = 0.017), high overall self-esteem (87.5 vs. 65.1%; p = 0.027), good school performance (75.0 vs. 51.2%; p = 0.036), and high social competence at age 5 (40.0 vs. 6.1%; p = 0.004). Maternal factors associated with a low degree of externalizing behaviours included current high social support (40.6 vs. 9.3%; p = 0.001), very good emotional health (50.0 vs. 25.6%; p = 0.029), and adequate good quality time spent with their child(ren) (78.1 vs. 46.5%; p = 0.006) (Table 3).

**Table 3:** Protective factors from problem behaviours in the presence of previous risk

	**Internalizing behaviours**			**Externalizing behaviours**		
	**Low degree (≤20th)**	**High degree (≥80th)**		**Low degree (≤20th)**	**High degree (≥80th)**	
	**N = 19**	**N = 50**	**p-value**	**N = 32**	**N = 43**	**p-value**
**Characteristics**	n (%)	n (%)		n (%)	n (%)	
Attends sporting events, art/cultural events, camping events	16 (84.2)	42 (85.7)	1	30 (93.8)	37 (86.0)	0.454
Spends time participating in activities	14 (77.8)	37 (74.0)	1	27 (87.1)	33 (76.7)	0.262
Low screen time - weekdays	12 (63.2)	29 (59.2)	0.764	22 (68.8)	21 (50.0)	0.105
Low screen time - weekends	4 (21.1)	7 (14.3)	0.486	7 (21.9)	6 (14.3)	0.395
Spends two or more days with friends outside of school	10 (52.6)	27 (54.0)	0.919	18 (56.3)	21 (48.8)	0.525
Two or more close friends	18 (94.7)	39 (78.0)	0.157	30 (93.8)	31 (72.1)	0.017
High maternal social support	4 (22.2)	7 (14.0)	0.464	13 (40.6)	4 (9.3)	0.001
High positive parenting interaction	5 (26.3)	8 (16.0)	0.326	8 (25.0)	5 (11.6)	0.13
High neighbourhood cohesion	1 (5.6)	5 (11.1)	0.664	8 (25.0)	3 (7.5)	0.052
At least 1 community involvement event	18 (94.7)	48 (96.0)	1	32 (100.0)	41 (95.3)	0.504
At least 1 school involvement event	17 (89.5)	39 (78.0)	0.491	28 (87.5)	37 (86.0)	1
Excellent or very good general health - child	18 (94.7)	41 (82.0)	0.264	29 (90.6)	37 (86.0)	0.724
High self-esteem - child	17 (89.5)	31 (63.3)	0.033	28 (87.5)	28 (65.1)	0.027
Well/Very well school performance	12 (63.2)	28 (56.0)	0.591	24 (75.0)	22 (51.2)	0.036
Excellent or very good emotional health - maternal	6 (31.6)	13 (26.0)	0.643	16 (50.0)	11 (25.6)	0.029
Quality time spent with children	12 (63.2)	29 (58.0)	0.697	25 (78.1)	20 (46.5)	0.006
High parenting sense of competence - efficacy	3 (20.0)	4 (11.4)	0.415	5 (25.0)	3 (9.4)	0.235
High parenting sense of competence - satisfaction	2 (13.3)	8 (22.9)	0.702	6 (30.0)	7 (21.2)	0.522
High child social competence	5 (33.3)	4 (11.4)	0.106	8 (40.0)	2 (6.1)	0.004
High parenting morale - 5 years	2 (13.3)	4 (11.4)	1	6 (30.0)	3 (9.1)	0.067
High maternal social support - 5 years	3 (20.0)	2 (5.9)	0.16	4 (21.1)	2 (6.1)	0.175

## Discussion

The findings of the present study confirm the importance of several recognized individual, family, and social factors in predicting the development of emotional and behavioural disorders [34], and build upon the results of the previous CPC follow-up studies at three and five years by demonstrating the persistent influence of early childhood adversity on developmental outcomes into the middle childhood years [24,25]. Broadly, the CPC research findings demonstrate the vital importance of maternal well-being and parent–child relationships on healthy development, particularly in the early years.

Although over 98% of children were reported to be in good to excellent general health, and despite the relatively high affluence of this sample of middle and upper income families with access to publicly funded universal health care, a substantial proportion of children (greater than 25% for each of the behaviour outcomes) were reported to exhibit problematic behaviours. These results highlight that the factors associated with an increased risk of behavioural disorders in children are not limited to conventional measures of socioeconomic status, as the largest number of vulnerable children reside in the middle class [14]. Further, because the emotional and behavioural problems identified in children and adolescents are dependent on the role of the reporting adult in their life, many problems may go undetected even by parents, suggesting that the occurrence of the problem behaviours reported here is likely an underestimate, particularly for internalizing behaviours [35,36]. An unexpected finding of the study was that the risk of both externalizing and internalizing behaviours was higher for males, a pattern not typically seen in previous research.

These results are consistent with previous research demonstrating strong relationships between early life events and internalizing and externalizing behaviours in adolescents and young adults [37,38]. Paramount among the significant factors in this study were indicators of maternal emotional and social well-being, including current and past maternal mental health risk, and several measures characterizing different facets of parenting difficulty. Notably, past maternal mental health risk, which captured distress in the prenatal and early postpartum periods, and has been linked to a reduced quality of parent–child relations [39], was associated with twice the risk of developing externalizing behaviours (31% vs. 14% in those with and without externalizing behaviours, respectively). Similarly, the majority of parenting difficulties measures reported on here (low positive parenting interactions, low parenting morale, low parenting sense of competence, and hostile parenting) were obtained at age 3 or 5 in the previous CPC follow-up studies. Thus, these findings denote the substantive continued influence of early parenting quality and maternal well-being into middle childhood and point to the potential value of timely intervention. Furthermore, it is interesting to note that different parenting variables were independently predictive of child behaviour outcomes, which suggests that they were measuring different aspects of the parenting environment, from parenting style to feelings of confidence.

Various models of resilience, or the ability to develop successfully in spite of adversity and environmental challenges, have been proposed to explain how risk and protective factors interact [40,41]. Resilience information is especially pertinent for preventive efforts, as recent evidence suggests that interventions enhancing protective factors may be more effective than those aimed at reducing risk of poor child outcomes [42]. Our research illustrates that certain child and maternal factors have a discernible protective effect against the development of problem behaviours, particularly those manifesting externally. Multiple resilience factors identified in the present study (high child self-esteem and social competence, high maternal social support and emotional health) are cogently related to adequate social support and connectedness, constructs which have been proposed to serve as a moderators between stressful events and poor mental health outcomes (including internalizing and externalizing behaviours) [43].

A sensitivity analysis that incorporated the middle range of scores on the behaviour scale into the low risk category was carried out, and the results with respect to protective factors were unchanged (data not shown). Of note, the cut-off used in the present study was based on the sample distribution of scores and the majority of children scored in the low range on both outcomes. Therefore, further examination of more stringent cut-offs are warranted, as are other approaches such as examination of interactions using the full sample in larger studies with both continuous and categorical outcomes.

Effective public health policies to prevent mental disorders and promote mental health “should encompass multiple preventive interventions addressing multiple causal trajectories for the relevant populations at risk” [16,44], demonstrating the need for both universal and targeted strategies. Because the demographic and social risks seen in this cohort are pervasive throughout social strata, programs which focus exclusively on low socioeconomic status or specific risk factors will miss a large number of children and families who are affected by adverse childhood experiences [14]. For example, although less than 5% of women reported household incomes of < $40,000 year, a large portion of the greater demographic risk observed at age eight was still related to food and/or income insecurity, with over 10% of mothers lacking adequate money for paying bills, obtaining food, or daily living expenses. Thus, our results support the assertion that to broadly develop resilience in the population, strategies for optimizing child development should begin early in life and should foster social support, resource management and coping strategies, and engagement with others and the community, regardless of socioeconomic status.

Although comprehensive identification of children and families that would benefit from targeted interventions remains a challenge, historically many of the most successful early life programs have been aimed at at-risk child populations [45]. Our results suggest that the early detection of mothers with parenting difficulties or a history of poor mental health, followed by provision of support that addresses sense of competence, morale, and parenting strategies, could lead to positive impacts on parental well-being and child mental and behavioural outcomes. Identification of mothers with punitive parenting styles and assistance with transitions to more supportive parenting have been associated with improvements in social and behavioural development and have been shown to buffer the effects of early adversities [46]. Development of personal skills that facilitate caring relationships are especially important for those with adverse life experiences as they may have more limited parenting knowledge, a point amplified because of the intergenerational persistence of parenting difficulties [47]. Similarly, interventions to develop better partner communication may reduce tension in relationships, leading to improved parenting competencies and reduced child maladjustment [48]. Such approaches would likely also capture those at risk of developmental problems [24], demonstrating the benefit of upstream approaches addressing this fundamental determinant of health.

Several limitations should be considered when interpreting the results of the present study. First, the sample for the CPC study was drawn from a population of women who received routine prenatal care in low risk maternity clinics. The relatively high level of education and income in this group potentially raises concerns about the generalizability of study results to those of lower socioeconomic status and to marginalized groups. Nevertheless, in earlier CPC follow-up studies it was found that 15% of children screened at highest risk for developmental problems, a proportion in line with expectations for a population-based setting and the risk and protective factors identified in this study cut across the socioeconomic spectrum. Additionally, the authors recognize that the absence of data on fathers in this study and many others regrettably perpetuates the substantial bias toward mother-child interactions that exists in the parenting literature. Further research that involves risk and resilience factors as they pertain to fathers is needed.

The original CPC study was a community-based study that was not initially designed for longitudinal follow-up, and traditional strategies to retain women were not immediately implemented. Women who were younger, had lower education and income, and were in poor physical health, were single or divorced, and who smoked were less likely to be represented in the follow-up CPC surveys [49]. These factors are similar to the characteristics of women who are generally difficult to retain in longitudinal research [50]. Retention strategies were implemented between the follow-up study at three, five, and eight years (e.g. routine contact, asking women to provide change in contact information). In all three studies, the participation rates were over 60%, and the women not retained in the cohort appear to be similar over the follow-up period. However, the potential for selection bias does exist given attrition of lower SES women across time (data not shown). If the demographic factors related to a lower likelihood of study participation adversely influenced child outcomes, this data will have underestimated the proportion of children with emotional and behavioural disorders. Therefore, our results are generalizable to populations sharing characteristics of the sample in the present study.

The dichotomous classification used in this study (internalizing/externalizing behaviours) is simplistic and does not capture all emotional and behavioural problems in children, but these two dimensions are most commonly used in research settings. The associations between parental well-being and the development of behavioural disorders in children are likely bidirectional, as the presence of emotional and behaviour problems in children may be a stressor for mothers and fathers, with subsequent influence on their mental health [51]. As well, although study outcomes were considered in isolation, this is an artificial distinction as children with externalizing disorders may have co-occurring internalizing disorders [52]. This category of children, as defined by scores at or above the 80th percentile for both internalizing and externalizing behaviours, composed 11% (51/444) of our study population. Similarly, the resilience factors identified reflect associations only, and due to the timing of assessment for some, we cannot preclude the possibility that the protective factors were manifestations of good mental health. Finally, because the study results were based on questionnaires, parents may have underestimated behavioural problems in their children, and it is not possible to determine if the children in whom parents reported problems have any psychiatric disorders, limiting assessment of the severity of health outcomes.

## Conclusions

Middle childhood problem behaviours were common in this sample of conventionally low-risk families. Adversity in critical periods of development was associated with internalizing and externalizing behaviours. However, individual and social resilience factors were shown to counter the influence of early misfortune on the likelihood of developing problem behaviours in middle childhood. Effective universal and targeted strategies to prevent mental disorders and promote mental health thus have the potential to produce substantial lifetime benefits in multiple wellness domains.

## Consent

Written informed consent was obtained from the patient’s guardian/parent/next of kin for the publication of this report and any accompanying images.

## Competing interests

The authors declare that they have no competing interests.

## Authors’ contributions

The study authors jointly conceived of and designed the study. JLC contributed to the interpretation of data and drafted the manuscript. SWM carried out the analysis of the data, contributed to the interpretation of data, and revised the manuscript for important intellectual content. SCT contributed to the interpretation of data and revised the manuscript for important intellectual content. All authors read and approved the final manuscript.

## Pre-publication history

The pre-publication history for this paper can be accessed here:

http://www.biomedcentral.com/1471-2431/14/166/prepub

## Supplementary Material

Additional file 1 **CPC 8 Questionnaire.** CPC-8 follow-up study questionnaire. Click here for file
