# Supplementary material for: Early childhood risk and resilience factors for behavioural and emotional problems in middle childhood
Source: BMC Pediatr. 2014 Jul 1;14:166. doi: 10.1186/1471-2431-14-166 (PMC4083129; doi:10.1186/1471-2431-14-166)
Supplement: Additional file 1 — CPC 8 Questionnaire. CPC-8 follow-up study questionnaire. [file 1471-2431-14-166-S1.pdf]

SECTION 1: LOOKING TO THE FUTURE

All children and families are different. There are no right answers to the following questions. We are interested in what you imagine for the future of your child, and what you perceive to be important for success.

1. What does your child want to be when he/she grows up?

____

2. Considering your child's educational future, what is the highest level of schooling that you think he/she will complete? (check one)

- ○ Complete post-graduate qualifications at a university (e.g. a Masters degree, professional designation such as law or medicine)
- ○ Complete a university degree
- ○ Complete a community college, technical college, or a trade/vocational program
- ○ Complete high school
- ○ Less than high school
- ○ No formal schooling

3. For each of the following, please rate its importance for your child's OVERALL SUCCESS IN THE FUTURE.

	Not important	Somewhat important	Neutral	Important	Very important
**Academic Skills: Math**	○	○	○	○	○
**Academic Skills: Science**	○	○	○	○	○
**Academic Skills: Reading/Writing**	○	○	○	○	○
**Technical Skills** (e.g. computers)	○	○	○	○	○
**Trade Skills** (e.g. electronics, carpentry, plumbing)	○	○	○	○	○
**Fine Arts** (e.g. theatre, music, art)	○	○	○	○	○
**Social Skills**	○	○	○	○	○
**Healthy Lifestyle** (e.g. good diet, adequate rest)	○	○	○	○	○
**Active Lifestyle** (e.g. fitness)	○	○	○	○	○
**Community Involvement** (e.g. volunteering)	○	○	○	○	○

SECTION 2: ACTIVITIES

This section asks about recreational and other activities that your family may participate in. Some of these questions refer to your child, while others are more specific to you, the parent.

1. Putting yourself in your child's shoes, what would you say is the best part of his or her week?

____

2. In an average year, HOW MANY TIMES does your child attend the following activities?

	None	Once	2 - 3 times	4 - 6 times	7 or more times
**A sporting event** (such as a hockey or football game)	O	O	O	O	O
**An arts or cultural event**	O	O	O	O	O
**A camp during school break**	O	O	O	O	O

3. In an average week, HOW MUCH TIME does your child spend participating in the following activities outside of school hours? Note: if activity is seasonal but has already occurred (e.g. outdoor soccer, dance classes in the spring), answer according to that season.

	None	Less than 30 min	30min - 1hr	1-2 hrs	2-4 hrs	4+ hours
**Individual recreational sports** (e.g. swimming, gymnastics, dance, martial arts)	O	O	O	O	O	O
**Team recreational sports** (e.g. hockey, soccer)	O	O	O	O	O	O
**Organized music/arts classes**	O	O	O	O	O	O
**A school club or after hours activity at school**	O	O	O	O	O	O
**Faith-based activities**	O	O	O	O	O	O
**Community-based clubs** (e.g. Cubs, Brownies)	O	O	O	O	O	O
**Tutoring**	O	O	O	O	O	O
**Unstructured or informal physical activities** (e.g., tag, pick-up soccer, playground play)	O	O	O	O	O	O

4. In general, how satisfied are you with your child's physical activity in an average week? (considering both formal and informal activities)

- O Very satisfied
- O Satisfied
- O Neutral
- O Dissatisfied
- O Very dissatisfied

5. Pick the TOP THREE REASONS why you choose activities for your child outside of school hours.

- ○ He or she likes it
- ○ To assist with child care
- ○ To have fun
- ○ To build a sense of self-worth
- ○ To be creative
- ○ Contributes to academic success
- ○ To be physically active
- ○ To build social relationships
- ○ To learn a skill
- ○ Part of my child's school curriculum
- ○ Cost
- ○ Provides mentorship from adults outside of your family
- ○ Other (specify): ____

6. Do you know about the 'Children's Fitness Tax Credit'? ($500 from Federal Government)

- ○ Yes
- ○ No
- ○ Not sure

If yes, did you claim the tax credit on your last income tax return?

- ○ Yes
- ○ No
- ○ Not sure

SECTION 3: THE TECHNOLOGY GENERATION

This section asks about your child's computer and media use.

1. **What is your child's favourite movie right now?** ____________________

2. **What is your child's favourite video game right now?** ____________________

3. **On average, how much time during a week day (Monday to Friday, OUTSIDE SCHOOL HOURS) and during a weekend day (Saturday or Sunday) does your child spend on the computer, watch TV/ DVDs/etc., or play video games?**

	None	Less than 30min/day	30min to 1hr/day	1-2 hrs/day	2-3 hrs/day	3 or more hrs/day	Don't Know
**Computer**							
Week day	O	O	O	O	O	O	O
Weekend day	O	O	O	O	O	O	O
**TV/DVD/Videos**							
Week day	O	O	O	O	O	O	O
Weekend day	O	O	O	O	O	O	O
**Wii**							
Week day	O	O	O	O	O	O	O
Weekend day	O	O	O	O	O	O	O
**Video games (e.g. Playstation, Nintendo DS)**							
Week day	O	O	O	O	O	O	O
Weekend day	O	O	O	O	O	O	O

4. **Please tell us about the most common activities that your child does on the computer.** (mark all that apply)
   - O Surfs the Internet
   - O Non-educational games
   - O Educational games
   - O Homework
   - O Social networking or socializing (e.g. Facebook or My Space, MSN Chat, Skype)
   - O Don't know
   - O My child doesn't use the computer
   - O Other (specify): ____________________

5. **To what extent are you involved in your child's choices concerning the above technologies?** (e.g. computer use, video games, etc.)

   O Not at all O Somewhat O Mostly O Always O My child doesn't use the above technologies

6. **Does your child have the following:**

A cell phone?
- O Yes
- O No

An iPod?
- O Yes
- O No

O **Any other device?** (specify): ____________________

SECTION 4: FAMILY, FRIENDS, AND COMMUNITY

This section asks about relationships and how you feel within and about your community.

The next few questions are about YOUR CHILD'S relationships with friends, family and others.

1. About how many days a week does your child do things with friends outside of school hours?

- Never
- 1 day a week
- 2-3 days a week
- 4-5 days a week
- 6-7 days a week

2. About how many close friends does your child have?

- None
- 1
- 2 or 3
- 4 or 5
- 6 or more

3. When it comes to meeting new children and making new friends, is your child:

- Very shy
- Somewhat shy
- About average
- Somewhat outgoing
- Very outgoing

4. During the past 6 months, how well has your child gotten along with other kids, such as friends or classmates? (excluding brothers or sisters)

- Very well, no problems
- Quite well, hardly any problems
- Pretty well, occasional problems
- Not too well, frequent problems
- Not well at all, constant problems

5. Since starting school in the fall, how well has your child gotten along with his/her teacher(s) at school?

- Very well, no problems
- Quite well, hardly any problems
- Pretty well, occasional problems
- Not too well, frequent problems
- Not well at all, constant problems

6. During the past 6 months, how well has your child gotten along with his/her parents?

- Very well, no problems
- Quite well, hardly any problems
- Pretty well, occasional problems
- Not too well, frequent problems
- Not well at all, constant problems

7. During the past 6 months, how well has your child gotten along with his/her brother(s)/sister(s)?

- Very well, no problems
- Quite well, hardly any problems
- Pretty well, occasional problems
- Not too well, frequent problems
- Not well at all, constant problems
- Not applicable (no brothers/sisters)

The following questions are about relationships and the support that YOU get from others.

	Strongly agree	Agree	Disagree	Strongly disagree
**8. If something went wrong, no one would help me.**	O	O	O	O
**9. I have family and friends who help me feel safe, secure and happy.**	O	O	O	O
**10. There is someone I trust whom I would turn to for advice if I were having problems.**	O	O	O	O
**11. There is no one I feel comfortable talking about problems with.**	O	O	O	O
**12. I lack a feeling of closeness with another person.**	O	O	O	O
**13. There are people I can count on in an emergency.**	O	O	O	O
**14. I feel part of a group of people who share my attitudes and beliefs.**	O	O	O	O
**15. There is no one who shares my interests and concerns.**	O	O	O	O

16. When I face problems or difficulties in my family, I respond by seeking information, advice, or support from: (mark all that apply)

- O Family member(s)
- O Co-worker(s)
- O Friend(s)
- O Neighbour(s)
- O A person who has faced a similar problem
- O Spiritual, religious or community leaders
- O Books, magazines or Internet sites
- O A health professional
- O Community agencies and programs designed to help families in similar situations
- O I don't seek information, advice or support from others
- O Other (specify): ______________________________

The following questions have to do with things that your child does and ways that you, as a parent, may react to him/her.

17. How often:

	Never	About 1/wk or less	A few times a week	1-2 times/day	Many times each day
...do you praise your child, by saying something like "Good for you!" or "What a nice thing you did! Thank you!", or "That's good going!"?	O	O	O	O	O
...do you and your child talk or play with each other, focusing attention on each other for five minutes or more, just for fun?	O	O	O	O	O
...do you and your child laugh together?	O	O	O	O	O
...do you do something special with your child that he/she enjoys?	O	O	O	O	O
...do you play sports, hobbies or games with your child?	O	O	O	O	O

The next questions are about certain aspects of your neighbourhood.

18. How do you feel about your neighbourhood as a place to raise your children? Is it:

○ Excellent? ○ Good? ○ Average? ○ Poor? ○ Very poor?

19. Are you involved in any local voluntary organizations such as school groups, church groups, community or ethnic organizations?

○ Yes

○ No

Please tell us whether you strongly agree, agree, disagree or strongly disagree with the following statements about your neighbourhood.

	Strongly agree	Agree	Disagree	Strongly disagree
**20. It is safe to walk alone in this neighbourhood after dark.**	○	○	○	○
**21. It is safe for children to play outside during the day.**	○	○	○	○
**22. There are safe parks, playgrounds and play spaces in this neighbourhood.**	○	○	○	○
**23. If there is a problem around here, the neighbours get together to deal with it.**	○	○	○	○
**24. There are adults in the neighbourhood that children can look up to.**	○	○	○	○
**25. People around here are willing to help their neighbours.**	○	○	○	○
**26. You can count on adults in this neighbourhood to watch out that children are safe and don't get in trouble.**	○	○	○	○
**27. When I'm away from home, I know that my neighbours will keep their eyes open for possible trouble.**	○	○	○	○

For the next questions, please think back over the last year.

28. In the last year, how often did you:

Attend or take part in a recreational event in your community? (e.g. a sporting event or concert)	○ Not at all	○ Occasionally	○ Frequently
Work with a children's group, club or team?	○ Not at all	○ Occasionally	○ Frequently
Help with a neighbourhood or community social event?	○ Not at all	○ Occasionally	○ Frequently
Help out a neighbour? (e.g. child care, yard work)	○ Not at all	○ Occasionally	○ Frequently
Go to a meeting dealing with community concerns? (e.g. Block Parents)	○ Not at all	○ Occasionally	○ Frequently
Go to neighbourhood events? (e.g. picnics)	○ Not at all	○ Occasionally	○ Frequently

SECTION 5: YOUR CHILD'S HEALTH

This section asks about your child's physical health, emotions and behaviours. We would like to know about your child's health, from HEAD to TOE!

1. In general, compared to other children the same age, would you say that your child's health is:

○ Excellent ○ Very good ○ Good ○ Fair ○ Poor

2. Over the past few months, how often has your child been in good health?

○ Almost all of the time

○ Often

○ About half of the time

○ Sometimes

○ Almost never

3. How much does your child currently weigh without shoes on?
(give your best estimate in pds or kgs)

|__|__|__| lbs. OR |__|__|__| kg.

4. How tall is your child without shoes on?
(give your best estimate in feet or cm)

|__| ft. |__|__| in. OR |__|__|__| cm.

The following question refers to conditions that have lasted or are expected to last 6 months or more and have been diagnosed by a health professional.

5. Has a health professional ever told you that your child has: (check all that apply)

○ Asthma

○ Food allergies

○ Other allergies (hay fever or allergic rhinitis)

○ Chronic (repeated) chest infections (bronchitis, pneumonia)

○ Chronic (repeated) ear infections (otitis media)

○ Cerebral palsy

○ Limited movement ability

○ Learning problems

○ Any other long-term conditions
IF YES, please specify:

____

○ Epilepsy

○ Lung problems

○ A hearing problem

○ A vision problem

○ Arthritis/joint problems

○ Kidney problems

○ Heart problems

○ Emotional, psychological, or behavioural problems

6. Has your child gone to see a doctor (GP) for a routine health exam in the last 12 months?

○ Yes ○ No

7. Has a health professional recently raised concerns about either of the following?

- ○ Your child being underweight
- ○ Your child being overweight
- ○ No concerns

8. In your opinion, do you feel your child:

- ○ Should weigh more?
- ○ Is about the right weight?
- ○ Should weigh less?

9. In the past year, have you seen or talked on the phone with any of the following specialists? (mark all that apply)

- ○ Speech and language pathologist
- ○ Pediatrician
- ○ Specialist pediatrician (e.g. respirologist, endocrinologist, neurologist)
- ○ Developmental pediatrician
- ○ Psychologist
- ○ Occupational therapist
- ○ Physiotherapist
- ○ Dietician
- ○ I have not had contact with a specialist
- ○ Other (specify): ________________________________

10. Was there ever a time during the past 12 months when you wanted to see a professional for your child but didn't?

- ○ Yes
- ○ No

If no, skip to question 11.

If yes, what type of professional did you want to see? (check all that apply)

- ○ Family doctor or medical specialist
- ○ Eye doctor
- ○ Public health nurse or other nurse
- ○ Dentist
- ○ Pharmacist
- ○ Naturopath
- ○ Psychologist or other counsellor
- ○ Social worker
- ○ Child Services
- ○ Physiotherapist
- ○ Chiropractor
- ○ Other (specify): ________________________________

If yes, what was the MAIN reason for not going? (check only one)

- ○ Too expensive
- ○ Didn't know who to see, where to go or who to call
- ○ Too far away or transportation problem
- ○ Couldn't get an appointment/takes too long
- ○ No hospital bed
- ○ Language problem
- ○ Too embarrassed to go
- ○ No one seemed to care
- ○ Past contacts were not helpful
- ○ Too busy
- ○ Help probably would not do any good
- ○ Other (specify): ____

11. Compared to other parents, have you ever felt that you were receiving inferior services for your child? (any service, not just the ones mentioned)

- O Yes
- O No

12. Has your child gone to see a dentist for a routine dental health exam in the last 12 months?

- O Yes
- O No

13. How many times per year do you schedule routine dental appointments for your child?

- O Once
- O Twice
- O 3 - 5 times
- O 6+ times
- O I don't schedule routine visits for my child

14. How old was your child when he or she first saw a dentist? |__|__| years old

15. Has your child ever had a cavity?

- O Yes
- O No
- O Don't know

If yes, how old was your child when he or she had their first cavity? |__|__| years old

16. How many days a week are your child's teeth brushed?

O 0 O 1 O 2 O 3 O 4 O 5 O 6 O 7

17. How many days a week are your child's teeth flossed?

O 0 O 1 O 2 O 3 O 4 O 5 O 6 O 7

18. In general, how long does it take your child to fall asleep?

- O 15 minutes or less
- O 16 minutes to 30 minutes
- O 31 minutes to 45 minutes
- O 46 minutes to 60 minutes
- O More than 60 minutes
- O More than 2 hours
- O More than 3 hours

19. How many total hours of sleep does your child get at night? |__|__| hours

Now some questions about certain experiences or difficulties that your child may experience at night.

20. Does your child have nightmares?

- O Yes
- O No   If no, skip to question 21.

If yes, how many times in a typical week does this happen? |__|__| times

21. Does your child snore during the night?

- O Yes
- O No
- O Don't know

22. Does your child wet his or her bed at night?

- O Yes   If no, skip to question 23.
- O No

If yes, how many times in a typical week does this happen? |__|__| times

23. Here is a list of statements about the feelings and behaviours of children. The response choices range from"Never or not true" to "Often or very true." Please tell us which response choice best describes your child within the past 6 months.

	Never or not true	Sometimes or somewhat true	Often or very true
Argues a lot with adults.	○	○	○
Blames others for own mistakes.	○	○	○
Easily annoyed by others.	○	○	○
Angry and resentful.	○	○	○
Temper tantrums or hot temper.	○	○	○
Does things that annoy others.	○	○	○
Gets back at people.	○	○	○
Swearing or obscene language.	○	○	○
Defiant, talks back to adults.	○	○	○
Can't concentrate, can't pay attention for long.	○	○	○
Jumps from one activity to another.	○	○	○
Interrupts or butts in on others.	○	○	○
Does dangerous things without thinking.	○	○	○
Doesn't seem to listen.	○	○	○
Fidgets.	○	○	○
Can't stay seated when required to do so.	○	○	○
Has difficulty playing quietly.	○	○	○
Impulsive, acts without thinking.	○	○	○
Has difficulty awaiting turn in games or groups.	○	○	○
Talks excessively.	○	○	○
Distractible, has trouble sticking to any activity.	○	○	○
Interrupts, blurts out answers to questions too soon.	○	○	○
Loses things.	○	○	○
Has difficulty following directions or instructions.	○	○	○
Needs to be told over and over that things are ok.	○	○	○
Worries about doing better at things.	○	○	○
Self-conscious or easily embarrassed.	○	○	○
Nervous, highstrung or tense.	○	○	○

Please tell us which response choice best describes your child within the past 6 months.

	Never or not true	Sometimes or somewhat true	Often or very true
Worries about past behaviour.	O	O	O
Worries about things in the future.	O	O	O
Unhappy, sad or depressed.	O	O	O
Has gained a lot of weight without trying to.	O	O	O
Gets no pleasure from usual activities.	O	O	O
Feels too guilty.	O	O	O
Cranky.	O	O	O
Has no interest in usual activities.	O	O	O
Has lost a lot of weight without trying to.	O	O	O
Can't sit still, restless or hyperactive.	O	O	O
Underactive, slow moving, or lacks energy.	O	O	O
Sleeps more than most children during the day and/or night.	O	O	O
Overtired.	O	O	O
Trouble sleeping.	O	O	O
Feels worthless or inferior.	O	O	O
Has difficulty making decisions.	O	O	O

24. Using the same rating scale as above, how often would you say that your child...

	Never or not true	Sometimes or somewhat	Often or very true
Shows sympathy to someone who has made a mistake?	O	O	O
Will try to help someone who has been hurt?	O	O	O
Volunteers to help clear up a mess that someone else has made?	O	O	O
If there is a quarrel or dispute, will try to stop it?	O	O	O
Offers to help other children (friend, brother or sister) who are having difficulty with a task?	O	O	O
Comforts a child (friend, brother or sister) who is crying or upset?	O	O	O
Spontaneously helps to pick up objects that somebody has dropped?	O	O	O
Will invite others to join in a game?	O	O	O
Helps other children (friends, brother or sister) who are feeling sick?	O	O	O
Helps those who do not do as well as he/she does?	O	O	O

25. Please rate your child's overall self-esteem using the following scale, where 0=very poor and 10 = very good. Give your rating by drawing a line through, or putting a notch on, the horizontal line.

0 ———————————————— **10**

Some children may experience events in their lives that cause them a great amount of worry or unhappiness.

26. Please indicate if (and when) any of the following events caused worry or unhappiness for your child. (check all that apply)

	**Younger than 5 years**	**5 years or older**
Death of parent(s)	O	O
Death in family (other than parents)	O	O
Divorce or separation of parents	O	O
Residential move (including within the city)	O	O
Stay in hospital	O	O
Stay in foster home	O	O
Other separation from parents for more than a few days	O	O
Someone moved into home	O	O
Someone moved out of home	O	O
Birth of sibling	O	O
Significant illness / injury of child	O	O
Significant illness / injury of a family member	O	O
Abuse / fear of abuse	O	O
Alcoholism or mental health disorder in family	O	O

SECTION 6: YOUR CHILD'S SCHOOL LIFE

This section asks about your child's school life in terms of what he or she is doing, how he or she is doing and what you think about his or her school.

1. **What part of going to school does your child enjoy the most?**

   ____

2. **What grade is your child in?** ____

3. **In which type of schooling is your child currently enrolled?** (check only one)
   - ○ Public or separate school
   - ○ Charter school
   - ○ Private school
   - ○ Home schooling

4. **Is your child enrolled in any of the following special academic programs?**
   - ○ School program for special needs students
   - ○ Language immersion program
   - ○ Specialized curriculum (e.g. arts, science, athletics)
   - ○ Not applicable
   - ○ Other (Please specify): ______________

5. **Did you delay your child's entry into school?** (e.g. your child was eligible for school but you decided to hold him/her back)
   - ○ Yes
   - ○ No   If no, skip to question 6.

   If yes, why?

   ____

6. **Has your child ever repeated a grade?** (including kindergarten)
   - ○ Yes
   - ○ No

7. **Has your child ever skipped a grade?** (including kindergarten)
   - ○ Yes
   - ○ No

8. **Has your child changed schools or type of schooling since starting school?**
   - ○ Yes
   - ○ No   If no, skip to question 9.

   If yes, how many times? ____ times

9. **Based on your knowledge of your child's school work to date (including report cards, if applicable), how is your child doing at school this year?**

   ○ Very well   ○ Well   ○ Average   ○ Poorly   ○ Very poorly

If your child is currently home schooled, please skip to section 7 - Your Health.

10. Does your child have an Individual Program Plan (IPP)?

- O Yes
- O No   If no, skip to question 11.

If yes, what are the reasons for the IPP? (mark all that apply)

- O Reading disorder or dyslexia
- O Attention Deficit and Hyperactivity Disorder (ADHD)
- O Fetal Alcohol Spectrum Disorder
- O Behavioural problems
- O Problem with speech or language
- O Other physical impairment/disability
- O Problem with sight
- O Medical or health problem
- O Problem with hearing
- O Mental illness/depression
- O Gifted/High IQ
- O English as an additional language
- O Learning difficulties
- O Autism, Asperger's syndrome, Autistic spectrum disorder or Pervasive Developmental Disorder
- O Other reason (specify): ______________________________

11. Which of the following factors were most important to choosing your child's school? (mark all that apply)

- O School is conveniently located
- O Child's friends attend same school
- O Siblings attend same school
- O School had good reputation
- O School has small class sizes
- O School caters to special needs
- O School offers specialized curriculum (e.g. arts, science, athletics, etc.)
- O Religious content and environment
- O School offers before or after school care
- O Attending this school makes it easier to get into a particular secondary school (feeder school)

12. During the previous school year, about how many days was your child absent from school for any reason?

- O None
- O 1 - 3 days
- O 4 - 6 days
- O 7 - 10 days
- O 11 - 20 days
- O More than 20 days
- O Not in school last year

13. What was the MAIN reason for your child being absent from school?
(select only one)

- ○ Health reasons (illness or injuries)
- ○ Problems with transportation
- ○ Problems with the weather
- ○ A family vacation
- ○ A fear of school
- ○ A problem with the teacher
- ○ A problem with children at school
- ○ Difficulties with childcare arrangements
- ○ Other (specify): ____________________

A few questions about what goes on at school.

14. How often does your child talk to you about what happens at school?

○ Every day ○ 3 - 4 times a week ○ 1 - 2 times a week ○ 1 - 2 times a month ○ Less than once a month ○ Never

15. Does your child tell you that they enjoy school?

○ Every day ○ 3 - 4 times a week ○ 1 - 2 times a week ○ 1 - 2 times a month ○ Less than once a month ○ Never

16. How often is your child upset or reluctant to go to school?

○ Every day ○ 3 - 4 times a week ○ 1 - 2 times a week ○ 1 - 2 times a month ○ Less than once a month ○ Never

17. To the best of your knowledge, how often has your child been bullied at school?

○ Every day ○ 3 - 4 times a week ○ 1 - 2 times a week ○ 1 - 2 times a month ○ Less than once a month ○ Never

18. To the best of your knowledge, how often has your child bullied other children at school?

○ Every day ○ 3 - 4 times a week ○ 1 - 2 times a week ○ 1 - 2 times a month ○ Less than once a month ○ Never

19. Do you or someone in your family do any of the following at your child's school?
(mark all that apply)

- ○ Help out in the classroom
- ○ Help out in the library
- ○ Help out on school trips
- ○ Help out at lunch time or recess
- ○ Help with fund-raising activities
- ○ Help with special events (e.g. sport days) or special interest groups like drama/sports
- ○ Participate in a parents' association/committee/group
- ○ Other (specify): ____________________

SECTION 7: YOUR HEALTH

You are important. We would like to know how you are feeling overall, and about certain experiences that you may have had.

This section asks for your views about your health.

1. In general, how would you rate your physical health in the past 6 months?

- Excellent
- Very good
- Good
- Fair
- Poor

2. In general, how would you rate your emotional health in the past 6 months?

- Excellent
- Very good
- Good
- Fair
- Poor

The next questions are about your outlook towards time use.

3. In the past 12 months, how often did you worry that you were not spending enough time with your spouse/partner?

- All of the time
- Most of the time
- Sometimes
- Never
- Not currently in a relationship

4. In the past 12 months, how often did you worry that you were not spending enough time with your child(ren)?

- All of the time
- Most of the time
- Sometimes
- Never

5. In the past 12 months, how often did you feel that the time spent with your child(ren) was good quality time?

- All of the time
- Most of the time
- Sometimes
- Never

6. In the past 12 months, how often did you worry that you were not spending enough time with your friends?

- All of the time
- Most of the time
- Sometimes
- Never

7. Raising children and finding the time and/or energy for things we need or want to do can be a challenge. How much tension would you say you feel from juggling all your activities of daily living?

O 0 O 1 O 2 O 3 O 4 O 5 O 6 O 7 O 8 O 9 O 10

No tension — A great deal of tension

8. Please indicate if any of the following has happened to you (or your spouse/partner, if applicable), <u>during the past 12 months.</u>

	Yes	No
Stopped full-time schooling.	O	O
Lost job or was unemployed.	O	O
Quit or retired from full-time work.	O	O
Started working or changed jobs.	O	O
Had financial problems.	O	O
Got married.	O	O
My spouse/partner and I separated.	O	O
Someone moved out of our home.	O	O
Someone moved into our home.	O	O
Arrival of a baby into our home.	O	O
Serious illness.	O	O
Serious illness of someone dear.	O	O
Death of someone dear.	O	O
Charged with any offence other than a traffic offense.	O	O

Now a few questions about things that may have happened when you were a child.

9. Before the age of 16...

	Yes	No
Did your parents separate or divorce?	O	O
Did you ever stay overnight at a detention or juvenile centre?	O	O
Did you ever stay overnight at a police station or jail?	O	O
Were you ever in foster care or a group home?	O	O

SECTION 8: BEFORE YOU GO

Finally, we would like to ask you some background questions about yourself. We use this information to compare groups of people, and not specific individuals.

1. Including yourself, how many people are living in your home?

Number of adults (18 and over): ____

Number of children (under 18): ____

2. What is your current marital status?

- ○ Single (never married)
- ○ Married
- ○ Common-Law (living with partner)
- ○ Divorced
- ○ Separated
- ○ Widowed

3. What was your marital status at the birth of your child?

- ○ Single (never married)
- ○ Married
- ○ Common-Law (living with partner)
- ○ Divorced
- ○ Separated
- ○ Widowed

4. How many changes in marital status have you experienced between the birth of your child and the present time? ____ change(s)

A 'change' can be ANY change, regardless of the type of change. For example, moving in with someone as common-law is a 'change,' as is a separation or divorce.

5. What is the highest level of schooling you have completed?

- ○ No formal schooling
- ○ Less than high school
- ○ Graduated high school
- ○ Some community college, technical college or trade/vocational program
- ○ Some university
- ○ Diploma or certificate from community college, technical college or trade/vocational program
- ○ Bachelor's or undergraduate degree
- ○ Master's degree
- ○ Degree in medicine or law
- ○ Earned doctorate

6. Which of the following best describes your <u>MAIN</u> activity? (check only one)

- ○ Employed or self-employed
- ○ A homemaker
- ○ Looking for employment
- ○ On paid maternity leave
- ○ A student
- ○ Other (specify): ______________________________

7. If employed or self-employed, about how many hours per week do you usually work?

- ○ Less than 10 hours
- ○ Between 10 and 19 hours
- ○ Between 20 and 29 hours
- ○ Between 30 and 39 hours
- ○ Between 40 and 49 hours
- ○ 50 hours or more
- ○ Not applicable (not currently employed

8. Which of the following was true for your household in the past 3 months?

	**True**	**Not true**
Sometimes we didn't have enough money for our food and daily living expenses.	○	○
We've gone to a food bank.	○	○
We have not been able to pay all of our bills.	○	○

9. Do you worry about whether the money you have will be enough to support your family?

○ Strongly agree ○ Agree ○ Disagree ○ Strongly disagree

10. How well would you say your household is managing financially these days?

- ○ We're living comfortably
- ○ We're doing alright
- ○ We're just about getting by
- ○ We're finding it quite difficult
- ○ We're finding it very difficult

11. What is your total income <u>before taxes and deductions</u> of all household members from all sources in the past 12 months?

- ○ Less than $20,000
- ○ $20,000 - $39,999
- ○ $40,000 - $59,999
- ○ $60,000 - $79,999
- ○ $80,000 - $99,999
- ○ $100,000 - $119,999
- ○ $120,000 - $139,999
- ○ $140,000 - $159,999
- ○ $160,000 or above
- ○ I prefer not to answer this question

12. What type of housing are you currently living in?

- House
- Apartment
- Duplex/Four-plex
- Group dwelling (e.g. hotel, shelter, boarding house, colony)
- Townhouse
- Condominium
- Other (specify): ______________________________

13. Do you rent or own the housing you are currently living in?

- Rent
- Own
- Living with family (no rent)
- Other (specify): ______________________________

14. How satisfied people are with the places they live depends on a lot of things - how much space they have, how hot or cold it gets, how quiet it is, how much privacy there is, and so on. Taking everything into account, please rate how satisfied you are with your home.

0	1	2	3	4	5	6	7	8	9	10
Completely dissatisfied										Completely satisfied

15. May we contact you if we have questions about this survey?

- No
- Yes

IF YES, please provide your <u>phone number</u> (optional): ______________________

and/or <u>email address</u> (optional): ______________________

16. May we contact you to participate in future follow-ups of the Community Perinatal Care Study?

- No
- Yes

IF YES, please provide your <u>phone number</u> (optional): ______________________

and/or <u>email address</u> (optional): ______________________

If you have any medical questions concerning your child, we encourage you to talk to your family doctor or call the Calgary Health Link at 403-943-LINK (5465). For urgent medical concerns please go the nearest Emergency Department. For mental health concerns please call the Distress Centre at 403-266-1606, the Calgary Counseling Centre at 403-265-4980, or your local Emergency Department.
